# Differences in anti-doping knowledge among Serbian professional athletes

**DOI:** 10.3389/fpubh.2025.1625859

**Published:** 2025-08-06

**Authors:** Zoran Vesic, Jelena Stojicevic, Nemanja Rancic, Gorica Milovanovic, Jelena S. Rasic, Nenad Radivojevic, Goran Prebeg, Dragan Atanasov, Milos Todorovic, Sonja Marjanovic, Milica Vukasinovic Vesic

**Affiliations:** ^1^Faculty of Political Sciences, University of Belgrade, Belgrade, Serbia; ^2^Military Medical Academy, Belgrade, Serbia; ^3^Antidoping Agency of Serbia, Belgrade, Serbia; ^4^University Clinical Center of Serbia, Belgrade, Serbia; ^5^Faculty of Sport and Physical Education, University of Belgrade, Belgrade, Serbia; ^6^Faculty of Physical Education and Sport Management, Singidunum University, Belgrade, Serbia; ^7^Faculty of Medicine of the Military Medical Academy, University of Defence, Belgrade, Serbia; ^8^Faculty of Sport, University “Union - Nikola Tesla”, Belgrade, Serbia

**Keywords:** anti-doping education, knowledge, athletes, sport, public health

## Abstract

**Objectives:**

The aim of this study was to examine the impact of anti-doping education among professional athletes on anti-doping knowledge.

**Methods:**

A prospective cohort study was conducted on differences in knowledge toward doping among 404 professional athletes in relation to their education about doping.

**Results:**

Participants who underwent education answered correctly significantly more often on most of the questions compared to participants without education [difference of about 20–30% in the rate of correct answers is in favor of participants with education on every question; 8.49 (SD 2.75) vs. 11.04 (SD 1.89); *p* < 0.001]. The majority of participants in the group with prior education against doping answered 10 or more questions correctly out of a total of 13, while the group without prior education against doping most commonly had 7 to 11 correct answers (*p* < 0.001). The most significant predictors of correct answers are gender, number of years of training, type of sport (individual or team sport), and prior education about doping. The largest contribution to this model comes from the variable “prior education against doping,” followed by the type of sport.

**Conclusion:**

Our research shows that prior anti-doping education is effective and has the essential contribution on athletes’ knowledge about doping.

## Highlights

- Prior anti-doping education is effective and has the essential contribution on athletes’ knowledge about doping.
- Participants who underwent anti-doping education answered correctly significantly more often on most of the questions compared to participants without education.
- More efforts should be made in the future to educate male athletes, as well as athletes in team sports, as they have shown less knowledge about doping.

## Introduction

Anti-doping education has become a critical component in the fight against doping in sports, as it aims to inform athletes about the risks and consequences of doping and to foster a clean sports environment. Effective educational programs are crucial in shaping athletes’ attitudes and behaviors toward anti-doping compliance. A growing body of research emphasizes the role of education in enhancing athletes’ knowledge and decision-making processes, as well as its potential to reduce doping violations. According to Listiani et al. (1), a systematic review that aims to review athletes’ knowledge of doping in sports to provide a foundation for evaluating anti-doping measures, particularly related to anti-doping education, demonstrated that athletes could have different level of understanding regarding anti-doping and highlighted importance of comprehensive anti-doping information (1). This work highlights the importance of further research to evaluate anti-doping education programs by assessing athletes’ knowledge as a crucial step toward enhancing the effectiveness of these programs and ensuring they are responsive to the evolving landscape of sports doping.

The World Anti-Doping Agency (WADA) (2) put education in a central focus of their anti-doping strategy. In its efforts to put education as a critical component in the fight against doping WADA developed Anti-Doping Education and Learning Platform (ADEL) (3), centralized platform offering educational solutions for athletes, athlete support personnel, Anti-Doping Organization (ADO) practitioners, researchers, and other members of the clean sport community (4).

All substances or methods included on the WADA prohibited list (The Prohibited List) meet at least two of the following three criteria: it improves or has the potential to improve sport performance; it poses a real or potential health risk to the athlete; and it violates the spirit of sport; as described in the 2021 World Anti-Doping Code (the Code) (5). The Prohibited List is a mandatory International Standard as part of the World Anti-Doping Program. The aim of the World Anti-Doping Program and the Code is to protect athletes’ right to participate in sports free from doping, thus promoting health, fair play, and equality among athletes worldwide (5, 6). Moreover, it seeks to ensure coordinated, effective, and harmonized anti-doping initiatives at both the international and national levels, focusing on detection, dettering and prevention of doping. In recent years, doping in sports has increasingly attracted the attention of medical, physiological, and scientific researchers (7). According to Gucciardi and colleagues (8), while medical and physiological researchers focus on improving detection methods (such as blood, urine, and gene tests) for the use of banned substances among athletes (9) researchers in the social sciences aim to better understand the psychosocial factors (such as attitudes, social environment, and beliefs) that may be critical for developing educational programs aimed at preventing such behavior (10).

Elite athletes are often reluctant to discuss the topic of doping with researchers, even when anonymity and confidentiality are guaranteed (11). This reluctance stems from the fact that they are asked to admit behaviors that could potentially jeopardize their careers (12). Nevertheless, in recent years, there has been a significant increase in research focusing on the attitudes, beliefs, and knowledge of elite athletes regarding doping and anti-doping regulations. However, most of these studies concentrate on athletes from Western countries (7, 13–15), while research involving athletes from Serbia remains relatively scarce.

While testing and research play a central and prominent role in WADA’s anti-doping strategy, its educational program is considered crucial for developing a lasting anti-doping culture in elite sports (3). Athletes are becoming more familiar with anti-doping rules, but there is still a noticeable lack of knowledge that should be addressed through appropriate programs.

The Knowledge, Attitude, and Practice (KAP) / Knowledge, Attitude, and Behavior (KAB) model was first developed in the 1950s. This model has been widely used in health education as a framework for understanding the mechanisms that influence changes in patients’ behavior and their health outcomes (16, 17). Since the 1990s, this research framework has been applied in the field of anti-doping (18). Findings from multiple studies consistently indicate that athletes’ positive attitudes toward doping are a significant predictor of increased susceptibility to its use. Athletes who express tolerant or justificatory views toward doping are considerably more likely to engage in risky behavior associated with the use of prohibited substances. (19–25). Furthermore, health concerns play a significant role in shaping attitudes toward doping (7). Awareness of the often negative health consequences of doping can contribute to the development of negative attitudes toward its use (26). At the same time, questions have arisen regarding the relationship between knowledge and behavior. Most anti-doping educational programs have a positive impact on increasing knowledge about doping, as well as shaping attitudes toward doping and reducing the likelihood of its use (18, 27).

The aim of this study was to examine the impact of anti-doping education among professional athletes on anti-doping knowledge.

## Methods

A prospective cohort study was conducted on differences in knowledge toward doping among professional athletes in relation to their education against doping. A total of 404 participants (or 96.65% of 418 potential participants who had to complete anti-doping education) took part in the study. The participants were recruited among scholarship athletes of The Athletics Federation of Serbia who must undergo mandatory anti-doping education.

The International Standard for Education 2021 (the ISE 2021) (2) defines high-priority groups (Registered Testing Pool (RTP) athletes and sanctioned athletes) as mandatory groups for education in the educational plan. In addition to the requirements of the International Standard, Anti-doping agency of Serbia (ADAS) defines additional mandatory groups for education in educational plan, according to Serbian national regulations. According to national regulations, athletes who receive national or city scholarships are required to pass two anti-doping educations during the financial year. Therefore, ADAS defines these groups of athletes as a high-priority group for whom anti-doping education is mandatory.

As in every year, ADAS conducted two anti-doping educations for scholarship athletes of The Athletics Federation of Serbia. The first anti-doping education was in the form of an electronic education, while the second was in person. The questionnaire, which is the subject of this research, was part of the first mandatory anti-doping education for scholarship athletes in 2023. Before the anti-doping education as the 30-min video form material, athletes were voluntarily able to fill out an anonymous survey. Anti-doping educational 30-min video material covered mandatory topics following the ISE 2021 requirements. Before listening to the video material, athletes had the opportunity to access the anti-doping questionnaire. Some athletes had the opportunity to complete anti-doping education earlier in their careers, while for a certain percentage of athletes the video material was their first anti-doping education. Athletes filled out the anonymous survey on the Moodle platform.

Based on the previous anti-doping education of the subjects, all subjects were divided into two groups: group with prior education against doping and group without prior education against doping.

The period for completing the questionnaire was from June 2023 to November 2023. Participation in this study was entirely voluntary. Completing the questionnaire indicated that the participant had given consent to participate in the research. It was emphasized that that the data collected from the study would be used solely for research purposes. This study was conducted in accordance with the principles of the Helsinki Declaration and was approved by Ethical Committee of the Anti-Doping Agency of Serbia (No decision 2025/1/1 on 22/04/2025; No administrative: 23–0422-2 on 22/04/2025).

The survey instrument was a structured questionnaire consisting of two sets of questions. The first set of questions collected sociodemographic data, such as gender, age, years of training, number of hours of weekly training, number of doping controls, level of education, level of competition, and type of sport (individual or team sport). The second set of questions analyzed the participants’ knowledge about doping through a questionnaire consisting of 13 questions.

The questionnaire used to measure knowledge created *ad hoc*. The internal consistency of the scale for the whole sample was *α* = 0.829 (good of reliability level). When looking at the Item-total Statistics, it can be seen that after deleting each of the 13 questions, Cronbach’s alpha ranges from 0.809 to 0.849.

The data were statistically processed using IBM SPSS, with the determination of measures of central tendency and variability. The Kolmogorov–Smirnov test was used to test the normality of the data distribution. The Mann–Whitney U test, Chi-square test, Student’s t-test for two independent samples, Spearman’s correlation analysis, and multivariate regression analysis were used to determine the statistical significance of the data at the *p* < 0.05.

## Results

A total of 404 participants took part in the study (160 women or 39.6%, 242 men or 59.9%, while two did not specify their gender – 0.5%). The average age of all participants was 22 years (IQR 19–26 years). Of all the participants, 82.2% or 332 had undergone education about doping, while only 72 or 17.8% had not received any form of education against doping.

The group of participants with prior education against doping was statistically significantly older than those who had not undergone education (median: 23 vs. 19; Table 1). There were significantly more women in the group with prior education against doping (43% vs. 23%). The participants who had underwent anti-doping education had significantly longer training periods and more hours spent on weekly training. They also had significantly more doping controls compared to those who had not received education against doping (average number of previous controls 3 vs. 1). Interestingly, in the group without prior education against doping, the majority of participants had a high school education (73.6%), while in the group with prior education against doping, there were significantly fewer with a high school education (56%). Those who had some form of education against doping participated more frequently in international competitions compared to those without prior anti-doping education (65.4% vs. 44.4%).

**Table 1:** Sociodemographic characteristics and basic information about training and competition of participants depending on prior education against doping.

Basic characteristics	Group without prior education against doping	Group with prior education against doping	*p* value
Age	19 (17–21.75)	23 (20–27)	<0.001*
Sex			
Women	17 (23.6%)	143 (43.1%)	0.007#
Men	55 (76.4%)	187 (56.3%)	
Not answered	/	2 (0.6%)	
Number of years of training	9.5 (5.25–12)	13 (10–17)	<0.001*
Number of hours of weekly training	12 (8–20)	16 (12–20)	0.001*
Number of previously doping controls	1 (1–2)	3 (1–10)	0.004*
Level of education			
Primary education	5 (6.9%)	9 (2.7%)	0.002#
Secondary education	53 (73.6%)	186 (56.0%)	
Higher education (vocational)	5 (6.9%)	27 (8.1%)	
Higher education (university)	9 (12.5%)	110 (33.1%)	
Level of competition			
International	32 (44.4%)	217 (65.4%)	<0.001#
National	24 (33.3%)	49 (14.8%)	
Both	16 (22.2%)	66 (19.9%)	
Type of sport			
Individual sports	42 (58.3%)	198 (59.6%)	0.943
Team sports	30 (41.7%)	134 (40.4%)	

*Mann–Whitney test; #Chi-square test; Data are presented as a number (percentage) or median with inter-quartile range.

If we analyze the participants’ knowledge about doping in relation to whether they had education against doping or not (Table 2), we can see that participants who underwent education answered correctly significantly more often on most of the questions compared to participants without education. Table 3 shows the rate of correct answers, and it can be observed that the difference in the rate of correct answers is in favor of participants with education on every question, with a difference of about 20–30%. The exception is the last question, “During doping control, does the athlete have the right to have a representative only if the doping control officer suggests it?,” where the low rate of correct answers among participants without anti-doping education is 30.6%, but also among participants with education (50.3%).

**Table 2:** Participants’ knowledge through a 13-question questionnaire depending on prior education against doping.

Questions	Group without prior education against doping	Group with prior education against doping	*p* value*
The list of banned doping substances is created and changed by WADA (Number 1)			
True	46 (63.9%)	310 (93.4%)	<0.001
False	4 (5.6%)	4 (1.2%)	
I do not know	22 (30.6%)	18 (5.4%)	
How often is the list of banned doping substances updated (Number 2)			
Every month	2 (2.8%)	13 (3.9%)	0.003
Annually	35 (48.6%)	208 (62.7%)	
Every 10 years	/	/	
Every 2 years	/	14 (4.2%)	
Every 5 years	/	4 (1.2%)	
It always stays the same	1 (1.4%)	12 (3.6%)	
I do not know	34 (47.2%)	81 (24.4%)	
Doping control (the sample collection process) can be conducted (Number 3)			
Only during training	2 (2.8%)	1 (0.3%)	<0.001
Only at the competition venue	17 (23.6%)	14 (4.2%)	
Anywhere	48 (66.7%)	315 (94.9%)	
I do not know	5 (6.9%)	2 (0.6%)	
Doping control (the sample collection process) can be conducted (Number 4)			
Immediately after the competition ends	10 (13.9%)	33 (9.9%)	<0.001
Before the competition starts	9 (12.5%)	6 (1.8%)	
Anytime	48 (66.7%)	291 (87.7%)	
I do not know	5 (6.9%)	2 (0.6%)	
Doping control (the sample collection process) is conducted (Number 5)			
At least one week after notification	3 (4.2%)	5 (1.5%)	<0.001
One day after notification	6 (8.3%)	11 (3.3%)	
Without advance notice	48 (66.7%)	306 (92.2%)	
I do not know	15 (20.8%)	10 (3.0%)	
The use of doping substances or methods are not punishable when used off-season (Number 6)			
True	4 (5.6%)	12 (3.6%)	<0.001
False	56 (77.8%)	308 (92.8%)	
I do not know	12 (16.7%)	12 (3.6%)	
Some supplements may contain substances banned in sports (Number 7)			
True	58 (80.6%)	306 (92.2%)	0.004
False	6 (8.3%)	16 (4.8%)	
I do not know	8 (11.1%)	10 (3.0%)	
Athletes can test positive for doping due to inadvertent doping (Number 8)			
True	46 (63.9%)	281 (84.6%)	<0.001
False	11 (15.3%)	35 (10.5%)	
I do not know	15 (20.8%)	16 (4.8%)	
Every athlete is solely responsible for every substance found in their body (Number 9)			
True	64 (88.9%)	319 (96.1%)	0.044
False	4 (5.6%)	7 (2.1%)	
I do not know	4 (5.6%)	6 (1.8%)	
When an athlete is sick, he/she can freely use any substance necessary for recovery (Number 10)			
True	9 (12.5%)	20 (6.0%)	<0.001
False	49 (68.1%)	294 (88.6%)	
I do not know	14 (19.4%)	18 (5.4%)	
If the treatment of an athlete’s health condition requires the use of a drug that contains substance from the Prohibited List, the following steps must be taken (Number 11)			
Visit a doctor, receive medical diagnosis report, and the prescription for appropriate therapy	25 (34.7%)	33 (9.9%)	<0.001
Fill out the TUE (Therapeutic Use Exemption) form and send it to the Anti-Doping Agency along with your medical documentation for approval	46 (63.9%)	297 (89.5%)	
There is no special action required. If the athlete needs a medication that can be bought without a prescription, it is enough to buy it at the pharmacy and start with a treatment in time	1 (1.4%)	2 (0.6%)	
If a doping control officer inform an athlete that she/he has been selected for doping control at an inconvenient moment, the athlete can arrange with the officer to carry out the control on another day, when the circumstances are more favorable (Number 12)			
True	11 (15.3%)	34 (10.2%)	0.007
False	45 (62.5%)	263 (79.2%)	
I do not know	16 (22.2%)	35 (10.5%)	
During doping control, the athlete has the right to have a representative only if the doping control officer suggests it (Number 13)			
True	27 (37.5%)	106 (31.9%)	0.003
False	22 (30.6%)	167 (50.3%)	
I do not know	23 (31.9%)	59 (17.8%)	

*Chi-square test; Data are presented as a number (percentage).

**Table 3:** Rate of correct answers depending on prior anti-doping education.

Rate of correct answers	Group without prior education against doping	Group with prior education against doping	*p* value*
Number 1	46 (63.9%)	310 (93.4%)	<0.001
Number 2	35 (48.6%)	208 (62.7%)	0.027
Number 3	48 (66.7%)	315 (94.9%)	<0.001
Number 4	48 (66.7%)	291 (87.7%)	<0.001
Number 5	48 (66.7%)	306 (92.2%)	<0.001
Number 6	56 (77.8%)	308 (92.8%)	<0.001
Number 7	58 (80.6%)	306 (92.2%)	0.003
Number 8	46 (63.9%)	281 (84.6%)	<0.001
Number 9	64 (88.9%)	319 (96.1%)	0.013
Number 10	49 (68.1%)	294 (88.6%)	<0.001
Number 11	46 (63.9%)	297 (89.5%)	<0.001
Number 12	45 (62.5%)	263 (79.2%)	0.003
Number 13	22 (30.6%)	167 (50.3%)	0.002

*Chi-square test; The order of questions is as in table number 2; Data are presented as a number (percentage).

**Figure 1:**
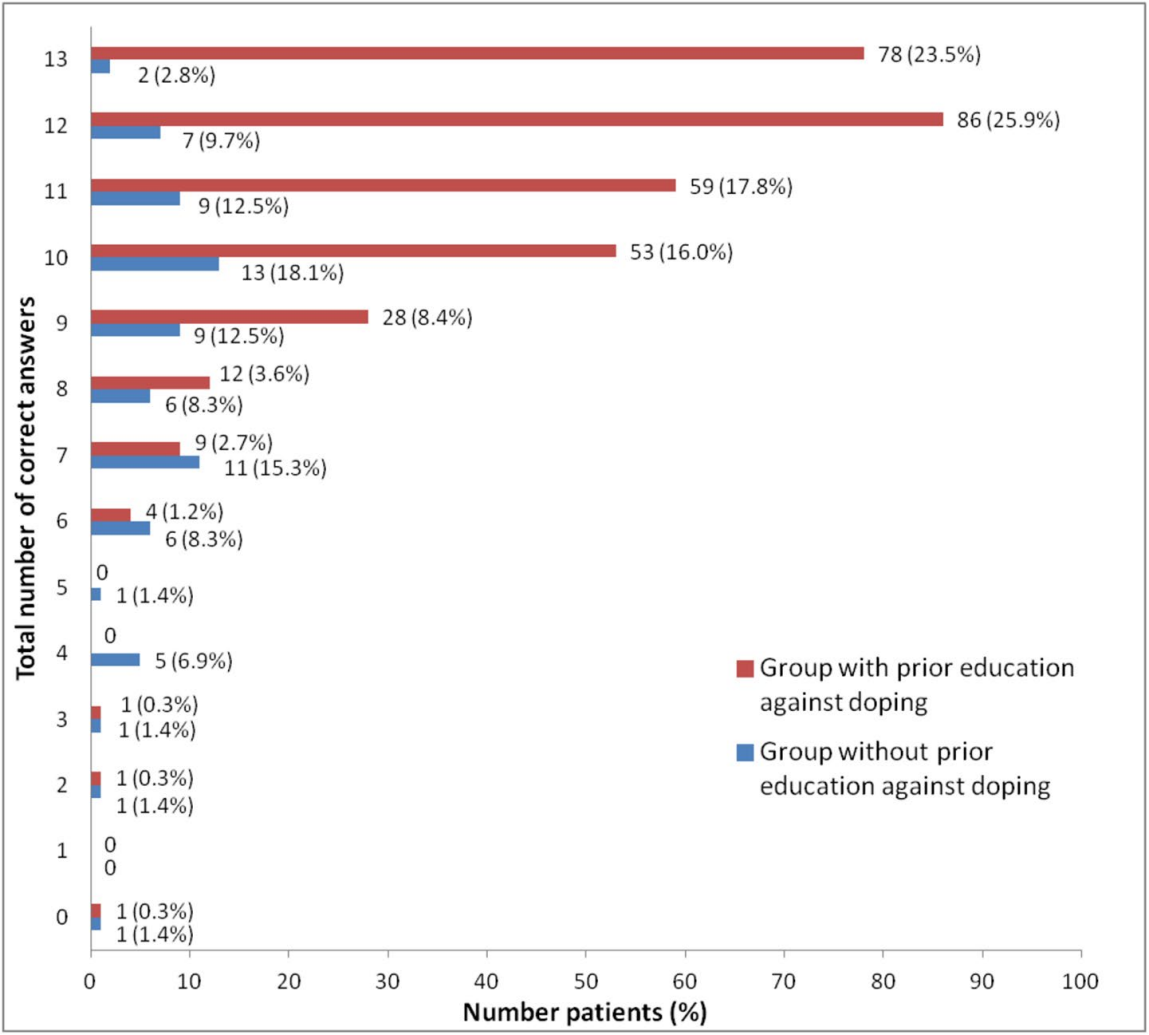
Distribution based on the total number of correct answers. Chi-square test, *p* < 0.001; data are presented as a number (percentage).

The average total number of correct answers per participant was 8.49 (SD 2.75) for the group without education, while for the group with education, it was 11.04 (SD 1.89), which is a statistically significant difference (Independent Samples Test; *p* < 0.001). The majority of participants in the group with prior education against doping answered 10 or more questions correctly out of a total of 13, while the group without prior education against doping most commonly had 7 to 11 correct answers (Chi-square test, *p* < 0.001; Figure 1).

If we analyze the correlation between the answers and the questions posed (Table 4), we can see that for all questions, there is a strong positive correlation between the correct answer and attending education about doping.

**Table 4:** Correlation between anti-doping education and knowledge questions related to doping in sports.

0 without education 1 with education 0 incorrect answer 1 correct answer		Have you gone through any kind of anti-doping education so far?
Number 1	*r*	0.349
	*p*	<0.001
Number 2	*r*	0.110
	*p*	0.027
Number 3	*r*	0.358
	*p*	<0.001
Number 4	*r*	0.219
	*p*	<0.001
Number 5	*r*	0.296
	*p*	<0.001
Number 6	*r*	0.192
	*p*	<0.001
Number 7	*r*	0.149
	*p*	0.003
Number 8	*r*	0.202
	*p*	<0.001
Number 9	*r*	0.124
	*p*	0.013
Number 10	*r*	0.219
	*p*	<0.001
Number 11	*r*	0.273
	*p*	<0.001
Number 12	*r*	0.150
	*p*	0.002
Number 13	*r*	0.151
	*p*	0.002

Spearman’s rho correlation.

Table 5 presents the multiple regression analysis, which shows that the variability in the responses to the posed questions can be explained by the model in 26.5% of the cases. The most significant predictors of correct answers are gender, number of years of training, type of sport, and prior education against doping. The largest contribution to this model comes from the variable “prior education against doping,” that could be expected, followed by the type of sport. Those who had prior education against doping also have a higher rate of correct answers. Participants competing in individual categories have a higher rate of correct answers compared to those competing in team sports.

**Table 5:** Predictors of the rate of correct answers.

Predictors	B	*p* value
Age	0.010	0.668
Sex	−0.439	0.031
Number of years of training	0.079	0.002
Number of hours of weekly training	0.028	0.063
Level of education	−0.065	0.585
Level of competition	−0.147	0.263
Type of sport	−0.618	0.004
Education about doping	2.011	<0.001

ANOVA, *p* < 0.001; R Square 0.265.

## Discussion

Doping is undeniably one of the greatest challenges facing the world of sports today (28), as it poses significant risks to athletes’ health, undermines the integrity of sports, and damages the legitimacy of elite competitions (29). To combat this issue, international and national organizations have implemented various anti-doping educational programs aimed to prevent athletes from violating anti-doping rules. These programs address among other both intentional and unintentional doping, encouraging athletes to adopt anti-doping practices such as reporting doping incidents and verifying whether dietary supplements have undergone serial testing for regulation breaches (15).

The International Standard for Education 2021 is a mandatory International Standard developed as part of the World Anti-Doping Program. The overall goal of the ISE 2021 is to support the preservation of the spirit of sport and to help foster a clean sport environment.

Education seeks to promote behavior in line with the values of clean sport and to help prevent athletes and other persons from doping. A key underpinning principle of the ISE 2021 is that an athlete’s first experience with anti-doping should be through education rather than doping control (5). The correlation between the answers and the questions posed indicates a strong positive correlation between the correct answer and attending education against doping, which is in line with the ISE requirements that an athlete’s first experience with anti-doping should be through education.

It is recognized that most athletes wish to compete clean, have no intention to use prohibited substances or methods and have the right to a level playing field (5). In addition to athletes, recent research and educational efforts have expanded to include athlete support personnel (ASP)—coaches, doctors, trainers, and family members—who play an important role in shaping the moral climate that influences athletes’ behavior (19). Studies reveal that ASPs, particularly coaches, often share similar knowledge gaps and lack adequate resources (30, 31). This can lead to a passive approach, where performance is prioritized over the integration of anti-doping education into their responsibilities (32).

Our findings indicate that participants who received prior anti-doping education performed better in knowledge assessments than those who did not. Specifically, the group with prior education against doping had the highest proportion of participants answering 10 or more questions correctly out of 13, while the group without prior education against doping predominantly scored between 7 and 11 correct answers (*p* < 0.001). Additionally, the group with prior education against doping had a significantly higher proportion of women (43% vs. 23%), longer training periods, more weekly training hours, and underwent more frequent doping controls compared to the group without prior education against doping. These findings are consistent with previous research by other authors as follows.

Hurst et al. (33) assessed the effectiveness of the UK Athletics “Clean Sport” anti-doping program among junior elite athletes. The program included a single 60-min session covering the WADC, anti-doping rule violations, doping control procedures, medication checks, and risks associated with sports supplements. Three months post-program, athletes demonstrated increased familiarity with anti-doping rules and a reduced likelihood of using prohibited dietary supplements (33). Similarly, García-Martí et al. (29) evaluated the Spanish Anti-Doping Commission’s (CELAD) program among 145 sports science students. Four months after completing the program, participants showed improved knowledge of banned substances and greater degree of moral disapproval of doping (15). These findings demonstrate the effectiveness of national anti-doping programs in enhancing knowledge about doping, reducing the intent to use prohibited dietary supplements, and fostering stronger moral judgment against doping practices.

In our study, significant predictors of correct answers included gender, years of training, participation in individual versus team sports, and prior anti-doping education. Educated participants scored higher overall, and those involved in individual sports outperformed their counterparts in team sports. These findings are in agreement with Lazuras et al.’s study of 750 elite Greek athletes, which found doping use to be more prevalent among individual-sport athletes (7.4%) (34). In this study was used an integrated social cognition model to examine the predictors of doping intentions in 1075 Greek adult elite-level athletes. Analyses showed that attitudes, normative beliefs, situational temptation, and behavioral control significantly predicted doping intentions. Additionally, Morente-Sanchez et al. reviewed doping attitudes and behaviors among elite athletes, noting that differences in doping tendencies between individual and team sports may stem from variations in sport-specific federation policies or discrepancies in the number and rigor of doping controls (e.g., more frequent testing in cycling compared to football) (7). The importance of anti-doping education can be estimated based on the study by Hurst et al. (15), where it can be seen that athletes who completed measures of doping susceptibility, intention to use dietary supplements, Spirit of Sport and moral values, anti-doping knowledge and practice, and whistleblowing, had after 3 months of education decreased doping susceptibility and intention to use dietary supplements coupled with increased importance of values, anti-doping knowledge, anti-doping practice and whistleblowing. In the other study by Hurst et al. (33), was evaluating UK Athletics’ Clean Sport program in preventing doping. In participants was measured knowledge of anti-doping rules, intention to use supplements, and supplement beliefs remained, as well as doping likelihood and moral disengagement at baseline, immediately after the program, and at 3-month follow-up. Compared to baseline, immediately after the program, participants had more knowledge about anti-doping rules and lower scores for intention to use supplements, beliefs about the effectiveness of supplements, doping likelihood and doping moral disengagement. At follow-up, knowledge of anti-doping rules, intention to use supplements, and supplement beliefs remained different from baseline, whereas doping likelihood and moral disengagement returned to baseline. After attending this UK program, participants were less likely to dope unintentionally and intentionally in the short term. However, the effects on intentional doping were not maintained after 3 months. These findings suggest that the program of anti-doping education reduces intentional doping in the short term, it needs to be strengthened to sustain effects in the long term. This can be achieved through continuous education.

Limitations: Our research has some limitations. Athletes typically attend anti-doping education programs biannually in Serbia. Athletes who received anti-doping education prior to the obligatory anti-doping education for the scholars 2023, could receive education by different anti-doping organizations, since our scholars (The Athletics Federation of Serbia) are mixed of national and international athletes. Content delivered by different anti-doping organizations could differ in effectiveness. Future research could aim to investigate whether different anti-doping education programs created by different anti-doping organizations (e.g., national versus international education course) could have difference in effectiveness. A methodological limitation was that the test was not conducted after the anti-doping education, but rather we conducted the test only before the anti-doping education. In future anti-doping educations, we will conduct testing of participants before and after the implemented education, in order to assess the success of the education.

## Conclusion

Our research shows that prior anti-doping education is effective and has the essential contribution on athletes’ knowledge about doping. Additionally, more efforts should be made in the future to educate male athletes, as well as athletes in team sports, as they have shown less knowledge about doping. Education programs should not only address athletes but also include coaches, physicians, and family members of athletes, as their relationship with athletes can either encourage or minimize doping behavior. Moreover, comprehensive curricula for sports education in schools should be revised to emphasize information about doping at an early stage, in order to raise awareness among amateur athletes as well.

## Data Availability

The raw data supporting the conclusions of this article will be made available by the authors, without undue reservation.
